# Spatially localized phosphorous metabolism of skeletal muscle in Duchenne muscular dystrophy patients: 24–month follow-up

**DOI:** 10.1371/journal.pone.0182086

**Published:** 2017-08-01

**Authors:** M. T. Hooijmans, N. Doorenweerd, C. Baligand, J. J. G. M. Verschuuren, I. Ronen, E. H. Niks, A. G. Webb, H. E. Kan

**Affiliations:** 1 Dept of Radiology, C.J. Gorter Center for High Field MRI, Leiden University Medical Center, Leiden, The Netherlands; 2 John Walton Muscular Dystrophy Research Centre, Newcastle University, Newcastle Upon Tyne, United Kingdom; 3 Dept of Neurology, Leiden University Medical Center, Leiden, The Netherlands; Rutgers University Newark, UNITED STATES

## Abstract

**Objectives:**

To assess the changes in phosphodiester (PDE)-levels, detected by ^31^P magnetic resonance spectroscopy (MRS), over 24-months to determine the potential of PDE as marker for muscle tissue changes in Duchenne Muscular Dystrophy (DMD) patients.

**Methods:**

Spatially resolved phosphorous datasets were acquired in the right lower leg of 18 DMD patients (range: 5–15.4 years) and 12 age-matched healthy controls (range: 5–14 years) at three time-points (baseline, 12-months, and 24-months) using a 7T MR-System (Philips Achieva). 3-point Dixon images were acquired at 3T (Philips Ingenia) to determine muscle fat fraction. Analyses were done for six muscles that represent different stages of muscle wasting. Differences between groups and time-points were assessed with non-parametric tests with correction for multiple comparisons. Coefficient of variance (CV) were determined for PDE in four healthy adult volunteers in high and low signal-to-noise ratio (SNR) datasets.

**Results:**

PDE-levels were significantly higher (two-fold) in DMD patients compared to controls in all analyzed muscles at almost every time point and did not change over the study period. Fat fraction was significantly elevated in all muscles at all time points compared to healthy controls, and increased significantly over time, except in the tibialis posterior muscle. The mean within subject CV for PDE-levels was 4.3% in datasets with high SNR (>10:1) and 5.7% in datasets with low SNR.

**Discussion and conclusion:**

The stable two-fold increase in PDE-levels found in DMD patients in muscles with different levels of muscle wasting over 2-year time, including DMD patients as young as 5.5 years-old, suggests that PDE-levels may increase very rapidly early in the disease process and remain elevated thereafter. The low CV values in high and low SNR datasets show that PDE-levels can be accurately and reproducibly quantified in all conditions. Our data confirms the great potential of PDE as a marker for muscle tissue changes in DMD patients.

## Introduction

Duchenne Muscular Dystrophy (DMD) is an X-linked disease caused by a mutation in the *DMD* gene which codes for the protein dystrophin. The absence of dystrophin in the muscle cells results in progressive muscle weakness and muscle damage which is reflected by changes in energy metabolism, inflammation, fibrosis and eventually by progressive replacement of muscle tissue by fat.[1] Although numerous clinical trials have so far led to conditional approval of a few compounds by the Food and Drug Administration (FDA) and European Medical Association (EMA), there is still an urgent need for more effective therapies and outcome measures in DMD.[2]

Quantitative magnetic resonance imaging (MRI) and spectroscopy (MRS) of muscle are becoming increasingly important as potential outcome measures for therapeutic evaluations in DMD, with both fat fraction (%fat) and water T2 being commonly used. [3] Therapy development aims to improve or preserve the quality of the muscle tissue. In order to inform about muscle tissue itself, a marker should reflect changes within the muscle and should have sufficient discriminative power. Unfortunately, neither %fat nor water T2 fulfill these criteria. While %fat correlates well with function, it reflects the replacement of muscle by fat and therefore does not allow any statement on muscle tissue itself. [4–6] Water T2 has a closer relation with muscle, as it reflects inflammation and/or edema-like processes in this tissue. However, it has a low discriminative ability in DMD due to the use of corticosteroids—the difference in water T2 from a corticosteroid-treated boy with DMD and a healthy control is close to the detection limit of any difference.[7]

Assessment of energy metabolism in muscle using phosphorous (^31^P) spectroscopy (MRS) has shown differences between healthy controls and DMD patients. [8–11]. As the concentration of the metabolites visible with ^31^P MRS is negligible in fat, this measurement almost exclusively measures muscle tissue. A broad variety of metabolic changes have been shown in DMD muscles, of which phosphodiesters (PDE) are especially interesting as a marker. PDE-levels are generally associated with membrane degradation products, and they are thought to reflect muscle membrane damage. [12] PDE levels have been shown to be elevated in the absence of increased fat%, and to remain elevated in more severely affected muscles in DMD. [13–15] They also revert back to normal after adeno-associated virus (AVV) vector therapy in Golden Retriever Muscular Dystrophy (GRMD) dogs, a canine model for DMD.[16] However, longitudinal assessments using ^31^P MRS in DMD are very limited and hence it is unknown how PDE changes over time in the same individual muscle.[14] Since muscles in DMD patients become affected at different time-points and at different rates it is essential to perform muscle specific measurements.[17, 18]

The overall aim of this work was to assess the time course of changes in phosphodiester (PDE)-levels detected by ^31^P MRS in individual muscles over a 2-year time period, using spatially resolved ^31^P MRS and quantitative Magnetic Resonance Imaging (qMRI) of lower leg muscles that represent different levels of muscle wasting. Because in DMD and many other muscular dystrophies muscle tissue is progressively replaced with fat, which directly reduces the SNR, we also aimed to assess the variability and accuracy of PDE-level quantification between two measurements. In addition, the effect of SNR on quantifying PDE-levels was assessed. Finally, the course of the Pi, PCr, ATP concentrations and intracellular tissue pH over two-year time-period were assessed with a post-hoc analysis. Our results show that PDE-levels were increased two-fold compared to healthy controls in muscles with different disease stages at virtually all time-points and did not change over a two-year period. The low CV and coefficient of reproducibility (CR) values in high and lower SNR datasets showed that PDE-levels can be accurately and reliably quantified in all conditions, which confirms the potential of PDE as an outcome measure.

## Methods

### Participants

DMD patients were recruited from the Dutch Dystrophinopathy Database [19] and all diagnoses were confirmed by genetic testing. A total of 18 DMD patients (mean: 9.8±2.6; range:5.5–15.4yrs.) and 12 healthy age-matched controls (mean:10.3±2.8; range:5-14yrs.) completed the baseline visit, 15 DMD patients (mean:10.2±2.9; range:6.5–16.4yrs.) and 10 healthy controls (mean:10.8±2.7; range: 5-15yrs.) completed the 12-month follow-up, and 12 DMD patients (mean:9.85±2; range:7.5–17.7yrs.) and 10 healthy controls (mean:12.1±2.6; range:5-16yrs.) completed the 24-month follow-up. Among the DMD patients, 13 were ambulant throughout the entire study, and 5 were wheelchair bound at baseline. All used corticosteroids with intermittent dosing regimens (varying between 8–10 days on/off). One of the DMD patients lost ambulation between the baseline and 12-month follow-up visit. The study was approved by the local medical ethical committee of Leiden University Medical Center (P12.214) and written informed consent was obtained from all patients and parents. Participant recruitment started June 2013.

In addition, for quality control purposes only, a total of 4 male healthy control subjects (age range: 33-82yrs.) were included in the study and scanned twice on the same day to assess reproducibility. All subjects gave written informed consent.

### MR examination

^31^P MRS datasets were obtained from the right lower leg using a 7T MR scanner (Philips, Achieva, Best, The Netherlands) with a custom-built double (^31^P and ^1^H) tuned volume coil. The subjects were positioned in a supine position with feet first into the scanner. The coil was placed at the thickest part of the calf directly distal to the patella, and scans were aligned with the tibia bone. The imaging protocol contained a 2D-Chemical Shift Imaging (CSI) dataset to assess energy metabolism (FOV 200x200/150x150 mm; matrix size 10 x 10; TR 2000 ms; samples 2048; FA 45°; Hamming weighted acquisition with 12 signal averages at the central k-lines), a B0 map as input for an image based shimming routine shimming (14 slices; slice thickness 8 mm, no slice gap; TR/TE 30/3.11ms; FA 20°; FOV 160x180 mm) and a T1-weighted sequence for anatomical localization (15 slices; slice thickness 7mm; interslice gap 0.5 mm; repetition time (TR) 10 ms; echo time (TE) 3.0 ms; flip angle (FA) 30°; FOV 180x200 mm). No slice selection was applied in feet/head direction for the 2D-CSI. As a result, ^31^P signal was measured over the full length of the coil, which covered 12 cm of the lower leg. The T1-weighted sequence was used to plan the 2D-CSI in such a way that one individual voxel was located within one muscle over the entire length of the coil, taking into account that the diameter and boundaries between individual muscles and muscle groups change along the length of the lower leg. (S1 File) The survey and the T1-weighted sequence were used as a reference to position the subject, and to plan the imaging stack as accurately as possible between the three subsequent time-points. On the same day, 3-point DIXON images (23 slices; slice thickness/gap 10/5mm; Repetition time(TR)/echo time (TE)/ΔTE 210/4.41/0.76 ms; 2 number of signal averages (NSA); flip angle 8°; 1x1x10mm) were acquired on a 3T MR system (Philips Ingenia, Best the Netherlands) with an anterior 16-element receive array and 12 posterior receive coils built into the patient table to quantify muscle fat fraction. The middle of the slice stack was positioned at the thickest part of the calf, to ensure accurate co-localization with the ^31^P data-set.

Finally, four HC subjects were scanned twice on the same day with the same imaging protocol and settings as described above for the ^31^P 2D CSI datasets. Subjects were repositioned in the scanner, as accurately as possible, in between the two measurements and the survey and T1w sequence were used as reference to plan the 2D CSI. CSI data sets were acquired as dynamic scans and retrospectively averaged to obtain datasets with 1 signal average for low SNR and one with 12 signal averages for high SNR.

### Data-analysis

All phosphorous data-sets were visualized with 3D chemical shift image package (3DCSi). Individual spectra were identified for six lower leg muscles: the lateral and medial head of the gastrocnemius (GCL and GCM), the peroneus (PER), the soleus (SOL), the tibialis anterior (TA) and tibialis posterior (TP) muscle, and exported as free induction decays. The FIDs were processed in the time domain using AMARES in the JMRUI software package (version 5, http://sermn02.uab.es/mrui/). [20] All metabolite signals were fitted with Gaussian line shapes, presented as a ratio over the γ-ATP or PCr signal, and corrected for T1 saturation effects using literature values.[21] In addition, the linewidth for PDE and β-ATP was set as described previously. [22] The shift in resonance between the Pi peak and PCr peak was used to calculate the intracellular tissue (pH = 6.75+log((3.27-S)/(S-5.69))). [23] Since muscles become progressively replaced by fat in DMD, several spectra in the DMD patients suffered from low SNR. SNR values were determined for each individual spectrum and noise was calculated as the standard deviation of the residual signal after fitting. Spectra with a SNR lower than 10:1 for PCr and the inability to identify all metabolite signals were excluded.

Datasets from the four healthy volunteers were used to calculate the CV and CR to assess the repeatability and accuracy of quantifying PDE-levels between the two repeated measurements in high SNR (HH) condition, low SNR (LL) condition, and between the high and low SNR condition of the first scan (HL): this latter comparison is to mimic the loss in SNR over time due to increased fatty infiltration. The CV was calculated by dividing the standard deviation of the repeated measures by the mean of the repeated measures. In addition, a Bland-Altman plot was computed to show the CR, which is defined as 1.96 times the standard deviation of the paired differences, for the same conditions (HH, LL and HL) as the CV values. The mean within-subject CV values and CR values are reported for all three conditions. In addition, PDE-levels were quantified from spectra acquired in the TA muscle of a single healthy subject with different number of averages (NSA = 1, 2,3,4,6 and12), resulting in varying SNR. Results were plotted as a function of the SNR of the PCr peak. The voxel was placed partly outside the leg in order to reach sufficiently low SNR. Similar analysis for other muscles was not feasible in practice because of the intrinsically higher SNR and the inability to place the voxel partly outside the leg. The 95% confidence interval of the plateau of the exponential fit was assessed to determine a SNR cut-off.

Fat and water images were reconstructed from 3-point Dixon images according to a six-peak fat model and used to calculate muscle fat fractions (SI) fat/(SI fat+ SI water))*100. (Hu et al 2008) The protocol was optimized to minimize T1 relaxation effects. The reconstruction did not account for T2* relaxation times. Regions of interest (ROI) were manually drawn for the six individual lower leg muscles on all the slices within the coverage of the 2D-CSI using Medical Image Processing Analysis and Visualization (MIPAV) software (http://mipav.cit.nih.gov). %Fat are presented as the mean value of all pixels within a ROI over multiple slices covering the same region as the 2D CSI.

### Statistical analysis

For the reproducibility assessment in healthy volunteers, a Spearman correlation was used to show the between-measurement reproducibility of PDE-levels for the three SNR comparisons. Subsequently, a Bland-Altman plot was used to assess the agreement between the two repeated measurements for HH, LL, HL.

For the primary analysis, differences in PDE-levels and %fat between healthy controls and DMD patients at baseline, 12-month follow-up and 24-month follow-up were assessed with a Mann-Whitney U test with correction for multiple comparisons (p<0.001). Thereafter, a Friedman test was used to assess differences in PDE-levels and %fat in DMD patients between the subsequent time-points. Here, the level of significance was set at p<0.01.

Post-hoc analyses were done to determine the relationship between PDE-levels and age in the TP muscle in DMD patients at baseline, using a Spearman correlation and for the difference in Pi, PCr, ATP and intracellular pH between patients and controls, as well as for the patients over time. We focused the analysis on the TP muscle as it was the only muscle which did not show differences in PDE-levels and %fat at baseline. The between-group analyses were done with a Mann-Whitney U test, and within the DMD group over time with a Friedman test (p<0.05). All statistical analyses were performed in SPSS version 20 for Windows (SPSS Inc., Chicago)

## Results

**Fig 1:**
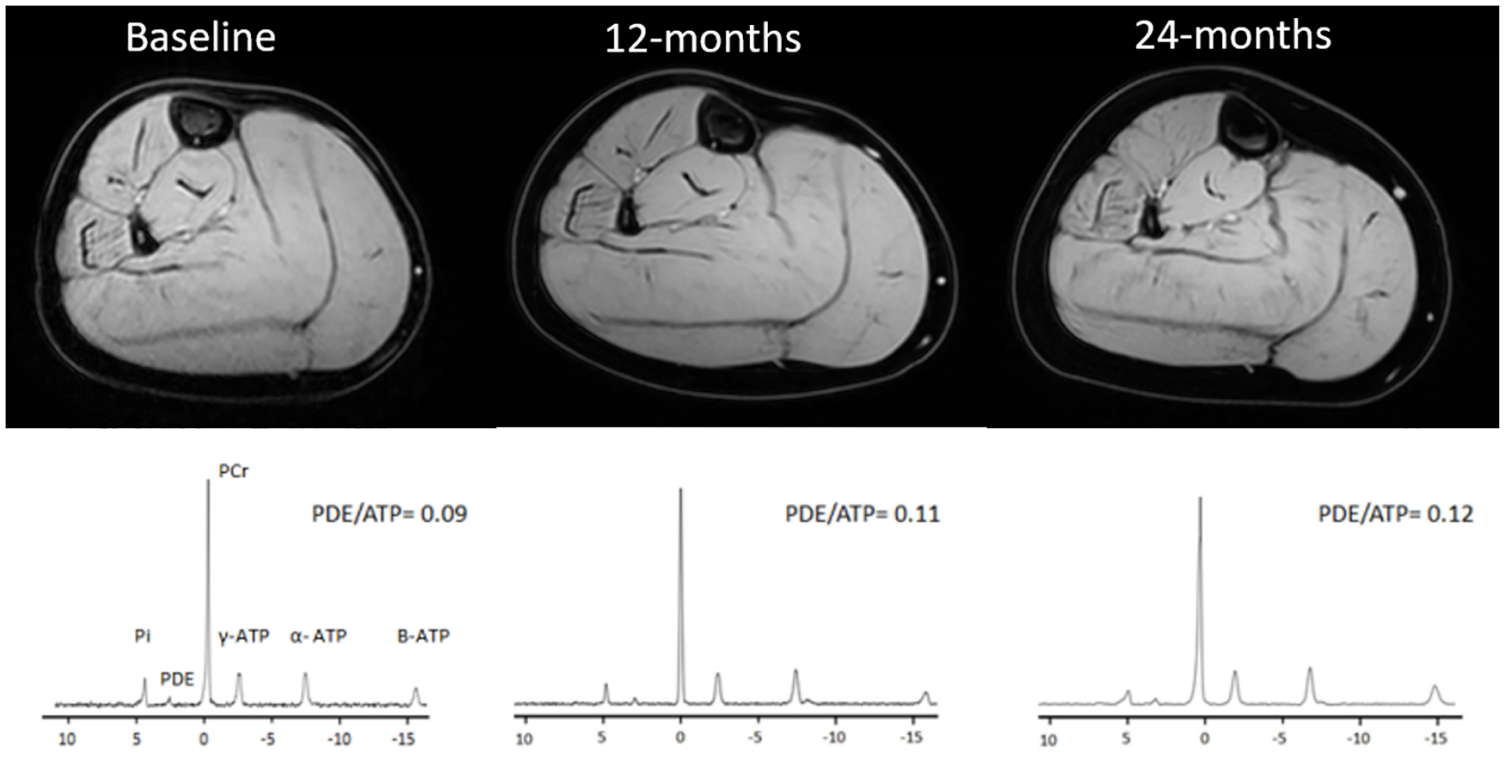
Representative reconstructed water images of the right lower leg and phosphorous spectra of the TP muscle of a DMD patient at baseline, 12-months and 24-months. PDE-levels in TP muscles are presented in the graph and %fat for the all analyzed muscle for all three time points are stated here: (Baseline GCL:5.6%;GCM:7.3%; SOL: 7.1%; PER:14,4%; TA: 6.24%; TP: 4.2%; 12-months: GCL: 6,6%; GCM:8.8%; SOL: 5.2%; PER:20.6%; TA: 5.71%; TP: 4.3%; 24-months: GCL:10.1%; GCM:11.3% SOL: 5.9%; PER:24.7%; TA: 7.3%; TP: 4.3%).

Phosphorous MR spectra and reconstructed water maps for a representative DMD patient for all three time points are shown in Fig 1. All spectra reached the quality control criteria for the healthy controls at baseline, five out of 60 spectra were excluded at 12-month follow-up and all spectra reached quality control at 24-month follow-up. For the DMD subjects 19 out of 108 spectra had to be excluded at baseline, 11 out of 90 at 12-month follow-up, and 4 out of 72 at 24-month follow-up. All of the spectra of the HC datasets obtained for the reproducibility assessment reached the quality control criteria.

### Repeated measures and effect of SNR

**Fig 2:**
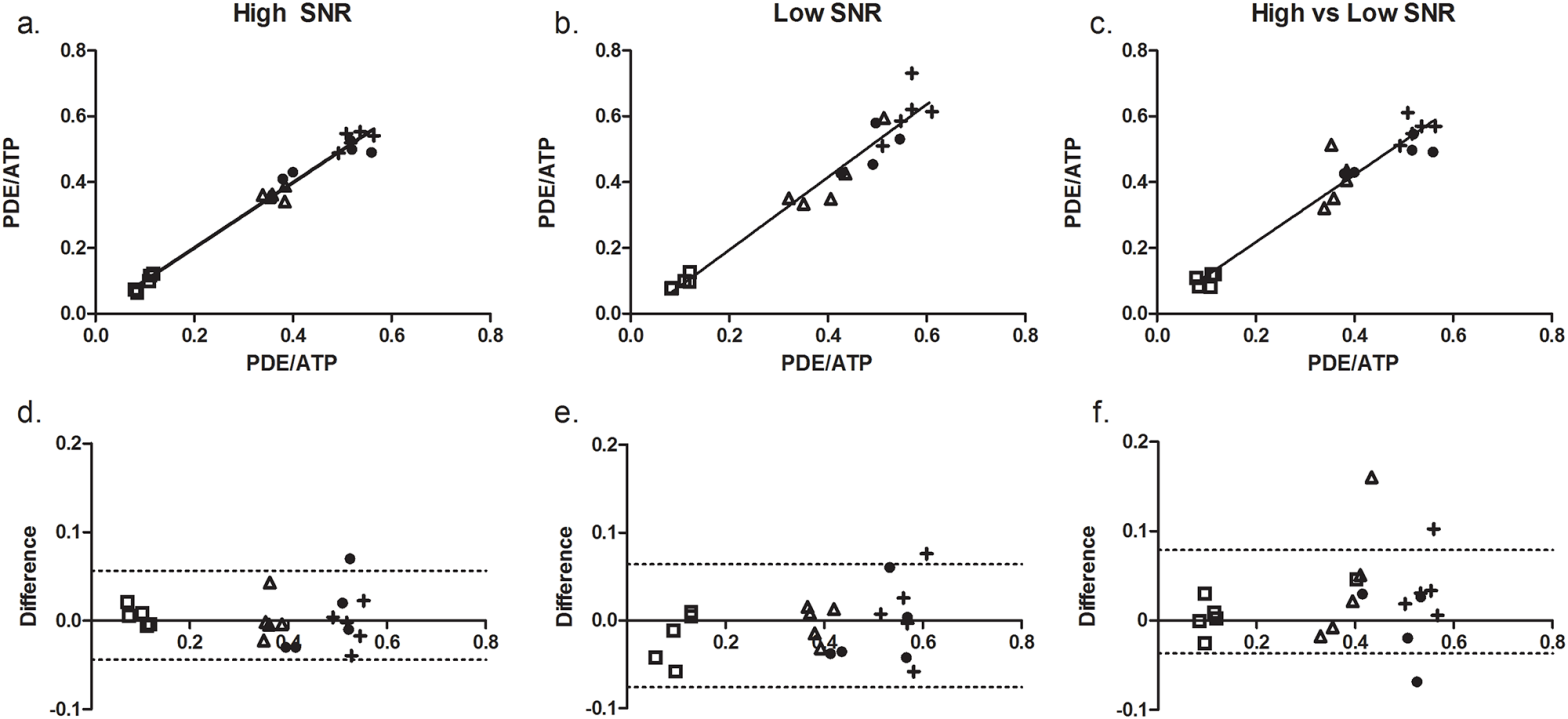
Intra class correlation coefficient between PDE-levels from the two repeated measurements and Bland-Altman plots of the difference in PDE-levels between the two repeated measurements for HH condition (a&d), the LL condition (b&e) and the HL condition (c&f). The five individual subjects are labelled with different symbols. Each symbol represents one individual muscle of an individual subject. The black line is the line of identity.

**Fig 3:**
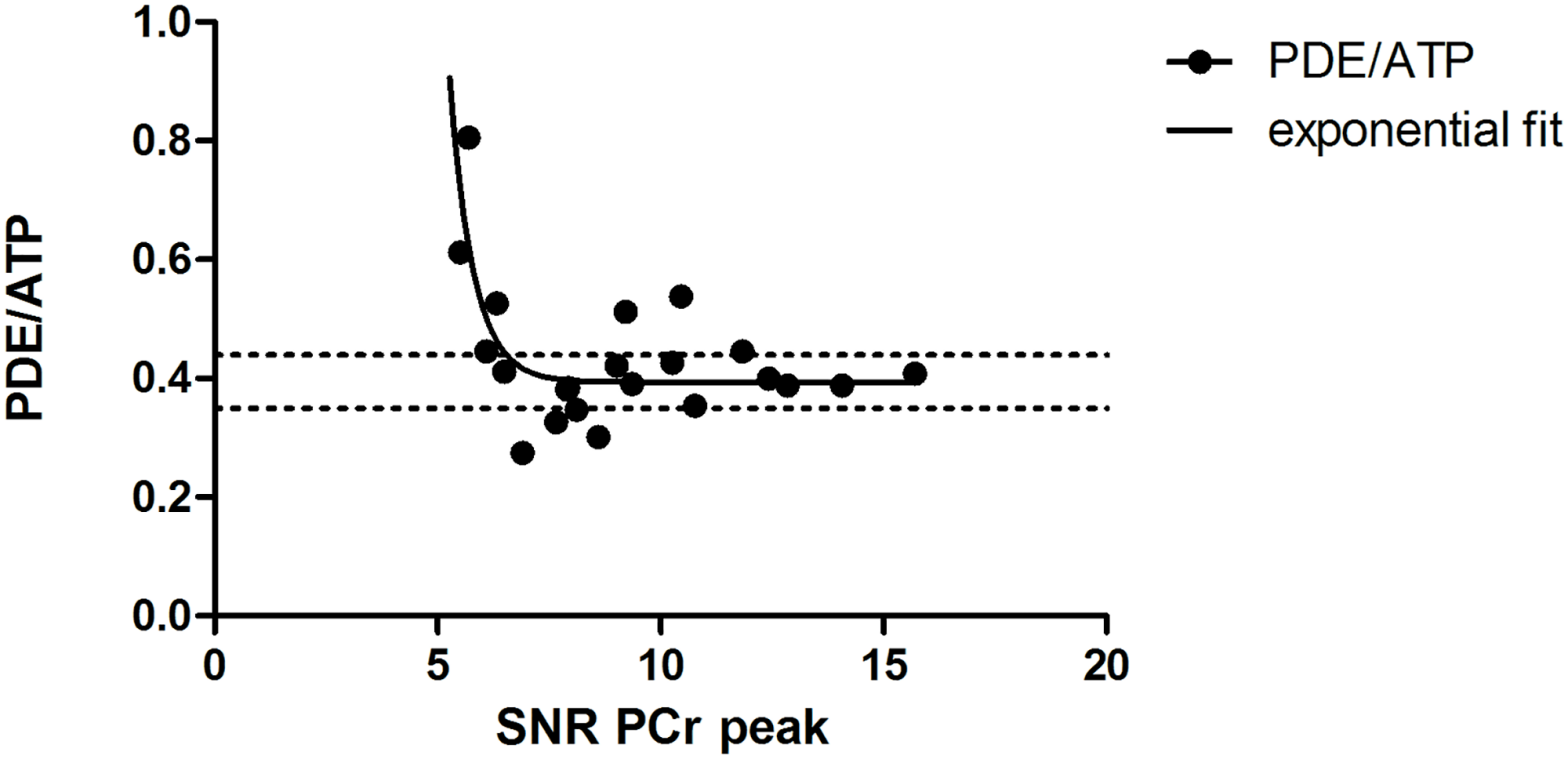
PDE/ATP as a function of SNR of the PCr peak. Each data point represents the PDE/ATP level assessed from the tibialis anterior muscle of the same subject under different SNR conditions. The black line represents the exponential fit and the dashed lines the 95% confidence interval of the estimated plateau.

To simulate intermediate fat infiltrated muscles, we generated low SNR datasets (NSA = 1, SNR = 15.2±4.1), and compared them to high SNR datasets (NSA = 12, SNR = 22.1 ±0.7). Quantification of PDE-levels proved to be highly reproducible in all three conditions with a mean within-subject CV of 4.3% (range:0.29–19%) in the HH condition (mean SNR: 22.1; range: 20.8–23.1), a mean within-subject CV of 5.7% (range:0.03–17.5%) in the LL condition (mean SNR: 15.2; range: 7.5–21.3), and a mean within-subject CV of 7.3% (range:0.73–26.3%) in the HL condition. This high reproducibility was also confirmed by intra-class correlation coefficients of 0.98 for the HH condition, 0.97 in LL condition and 0.93 in HL condition and good agreement in the Bland Altman plots. (Fig 2) In addition, we found that PDE-levels were overestimated at lower SNR levels of the PCr peak in the TA muscle (Fig 3). Based on the exponential fit, it seems that PDE-levels can be quantified within 95% of the confidence interval above SNR levels of 10.4:1 for the PCr peak in the TA muscle.

### %fat and PDE-levels

**Fig 4:**
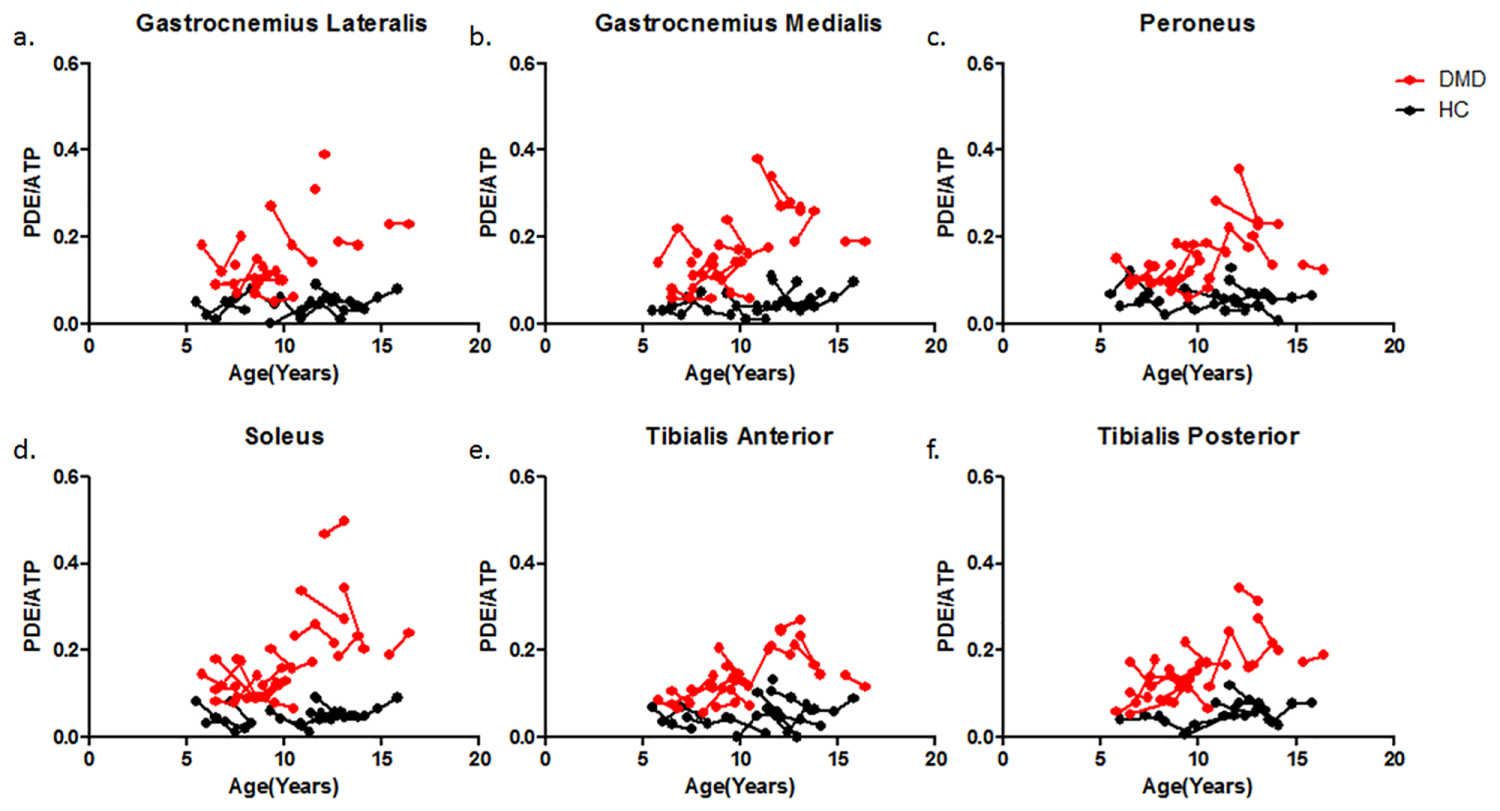
Individual trajectories of PDE-levels plotted as a function of age in DMD patients (Red) and HC subjects (Black) for the GCL (a), GCM (b), PER (c), SOL (d), TA (e) and TP (f) muscle. The dots with a connecting line represent an individual subject. PDE-levels were significantly different between groups but did not change over a two-year time period.

**Fig 5:**
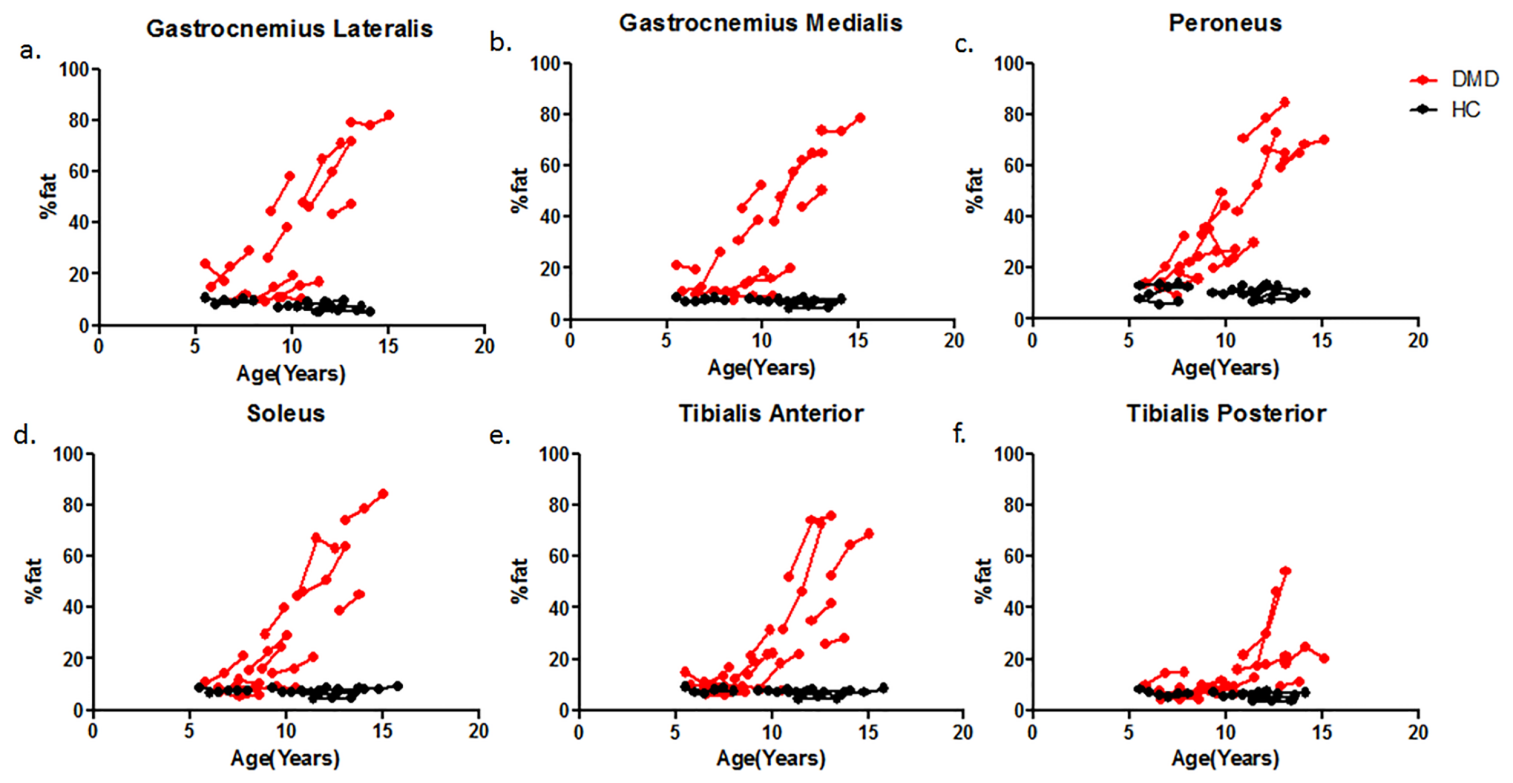
Individual trajectories of fat fraction plotted as a function of age in DMD patients (Red) and HC subjects (Black) for the GCL (a), GCM (b), PER (c), SOL (d), TA (e) and TP (f) muscle. The dots with a connecting line represent an individual subject. Fat fractions were significantly increased compared to controls in all muscles and all time points with the exception of the TP and TA muscle.

PDE-levels were significantly elevated compared to healthy controls at all time-points for all individual leg muscles (p<0.001), with the exception of the TP muscle at baseline (p = 0.004). No differences were found in PDE-levels between baseline, 12-month follow-up and 24-month follow-up for the individual muscles (p>0.01). (Fig 4) However, post-hoc analysis showed a significant positive correlation with age in the TP muscle at baseline (R = 0.79 p = 0,01). This was also the only muscle in which fat fraction was not significantly elevated. In addition, %fat significantly increased over time for the GCM, GCL, SOL and PER muscle. No differences in %fat were detected between time-points for the TA (p = 0.061) and the TP muscle (p = 0.097). (Fig 5)

### Post-hoc analysis for the other metabolic indices

**Fig 6:**
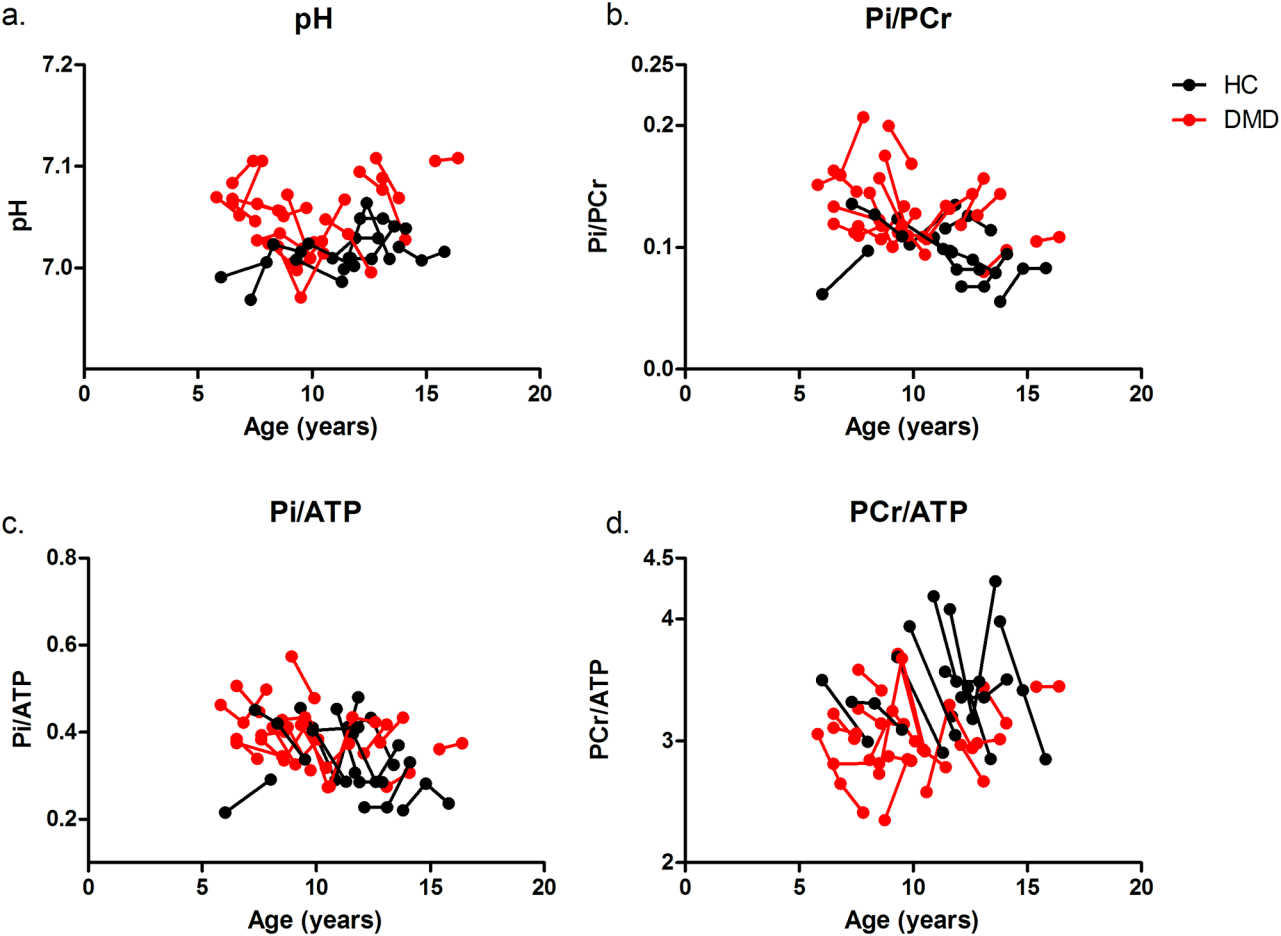
Individual trajectories of Pi/PCr (a), pH (b), PI/ATP (c) and PCr/ATP (d) plotted as a function of age in DMD patients (Red) and HC subjects (Black) for the TP muscle. The dots with a connecting line represent an individual subject. Note the inconsistent changes in PCr/ATP and the more pronounced changes above the age of 10 years.

Mean and standard deviation of the metabolic indices in the DMD patients and HC subjects at the three time points are shown in Table 1. The individual trajectories of the metabolic indices and pH as a function of age in the TP muscle are shown in Fig 6. Intracellular tissue pH was significantly elevated compared to controls in all analyzed muscles at baseline, 12-month follow-up and 24-month follow-up, with the exception of the TP muscle at 12-month and 24-month follow-up. Pi/PCr was significantly increased compared to healthy controls in all muscles at baseline, in the SOL and PER muscle at 12-month follow-up and in the SOL, PER, TP and GCL muscle at 24-month follow-up. In addition, elevated Pi/ATP was found in the SOL and PER muscle at baseline, in the SOL and GCM at 12-month follow-up and in all muscles except the TA muscle at 24-month follow-up. Finally, PCr/ATP was significantly decreased compared to controls in the PER, TP, and TA muscle at baseline. No differences were found between groups at the subsequent time points. None of the metabolic indices changed over the three time points. However, more pronounced changes, although not at a statistically significant level, were visible at 24-month follow-up compared to the other time-points.

**Table 1:** Mean values ±SD for the Pi/PCr, Pi/ATP, PCr/ATP and intracellular tissue pH in healthy controls and DMD.

		DMD			HC	
**pH**	Baseline	12months	24months	Baseline	12months	24months
GCL	7,07±0,04*	7,06±0,04*	7,06±0,02**	7,03±0,03	7,02±0,02	7,01±0,01
GCM	7,07±0,04*	7,07± 0,05*	7,08±0,04**	7,03±0,03	7,02±0,02	7,01±0,02
PER	7,06±0,03**	7,05±0,04*	7,05±0,03**	7,01±0,02	7,02±0,03	7,00±0,01
SOL	7,08±0,05*	7,06±0,05*	7,07±0,03**	7,03±0,02	7,02±0,02	7,01±0,01
TA	7,05±0,03**	7,05±0,03*	7,04±0,03**	7,003±0,02	7,001±0,03	6,97±0,02
TP	7,06±0,03**	7,05±0,04	7,05±0,03	7,008±0,02	7,02±0,02	7,01±0,02
**Pi/PCr**						
GCL	0,14±0,05*	0,12 ±0,05	0,11±0,02*	0,11±0,02	0,09±0,02	0,09±0,02
GCM	0,16±0,04*	0,13±0,02	0,13±0,05	0,12±0,03	0,11±0,02	0,11±0,02
PER	0,14±0,03*	0,14±0,02*	0,14±0,04*	0,10±0,03	0,11±0,02	0,09±0,02
SOL	0,14±0,03**	0,13±0,04**	0,13±0,03*	0,10±0,016	0,11±0,02	0,10±0,02
TA	0,15±0,04*	0,13±0,03	0,12±0,02	0,11±0,02	0,12±0,03	0,10±0,02
TP	0,13±0,03*	0,13±0,02	0,14±0,03*	0,09±0,03	0,10±0,02	0,09±0,04
**Pi/ATP**						
GCL	0,43±0,09	0,36±0,11	0,36±0,07*	0,35±0,09	0,30±0,06	0,31±0,06
GCM	0,46±0,08	0,38 ±0,07*	0,44±0,13*	0,4±0,11	0,32±0,06	0,34±0,06
PER	0,43±0,11*	0,39±0,08	0,42±0,09*	0,38±0,07	0,34±0,08	0,30±0,05
SOL	0,46±0,09**	0,37±0,09**	0,42±0,09*	0,35±0,07	0,32±0,06	0,32±0,06
TA	0,43±0,09	0,37±0,06	0,38±0,09	0,38±0,07	0,37±0,12	0,34±0,06
TP	0,39±0,07	0,39±0,06	0,39±0,06*	0,35±0,10	0,33±0,07	0,27±0,11
**PCr/ATP**						
GCL	3,15±07	3,0±0,56	3,37±0,2	3,35±0,33	3,09±0,38	3,3±0,31
GCM	3,22±0,45	2,89±0,44	3,38±0,35	3,47±0,42	2,96±0,37	3,36±0,24
PER	3,13±0,37*	2,82±0,31	3,21±0,41	3,6±0,39	3,18±0,48	3,34±0,26
SOL	3,23±0,4	2,87±0,45	3,28±0,28	3,34±0,39	2,98±0,41	3,4±0,22
TA	2,97±0,38*	2,86±0,3	3,23±0,3	3,56±0,29	3,16±0,36	3,36±0,41
TP	3,02±0,37*	3,09±0,31	2,94±0,27	3,68±0,34	3,27±0,26	3,21±0,47
**PDE/ATP**						
GCL	0,17±0,09^#^	0,13±0,07^#^	0,13±0,04^#^	0,05±0,03	0,05±0,02	0,04±0,02
GCM	0,17±0,09^#^	0,17±0,09^#^	0,16±0,07^#^	0,06±0,03	0,04±0,01	0,06±0,03
PER	0,16±0,08^#^	0,13±0,06^#^	0,14±0,04^#^	0,06±0,03	0,05±0,03	0,06±0,02
SOL	0,17±0,11^#^	0,15±0,11^#^	0,14±0,07^#^	0,06±0,02	0,05±0,01	0,04±0,03
TA	0,14±0,06^#^	0,12±0,06^#^	0,14±0,05^#^^#^	0,06±0,03	0,05±0,02	0,03±0,03
TP	0,14±0,08	0,16±0,06^#^	0,15±0,11^#^	0,07±0,03	0,07±0,02	0,05±0,02

Significant differences between patients and controls are marked with:

* for p<0.05,

** for p<0.0005, and

^#^ for p<0.001.

Pi = inorganic phosphate, PDE = phosphodiester, PCr = phosphocreatine, ATP = adenosine triphosphate GCL/GCM = lateral and medial head gastrocnemius, PER = peroneus, SOL = soleus, TA = anterior tibialis

## Discussion

### Repeatability assessment

In this study, we assessed the accuracy and reproducibility of quantifying PDE-levels by using spatially resolved ^31^P MRS data in low and high SNR conditions in healthy volunteers. Our data proved that quantification of PDE-levels is highly reproducible between measurements in both the HL, LL and HH conditions, which is reflected by low CV values, high intra-class correlations, and good levels of agreement. As the number of studies assessing the reproducibility of resting energy metabolites in muscle is limited, comparison to literature values is challenging. However, they are in line with the reproducibility values of different metabolic markers and exchange rate constants using dynamic ^31^P MRS in healthy controls, where CV values ranged between 8–11% for phosphate concentrations, between 4 and 12% for PCr recovery and between 3.4 and 20% for exchange rate constants of PCr-to-ATP [24–26]. Generally, CV values of 10% or less are considered to be an acceptable level of agreement between measurements [27]. Previous studies assessed the reproducibility of %fat, cross-sectional area (CSA) and water T2 measurements, commonly used MR imaging measures in DMD, in both HC subjects and DMD patients within and between centers. CV values ranging from 0.8–8% were reported within centers with a mean CV of 4.9%, 3.1% and 3.7% for %fat, CSA and T2 relaxation times [28–30]. CV values of 1.8–7.2% were reported across centers with a mean CV of 7.2, 3.4 and 1.8 for %fat, CSA and water T2 relaxation times (MRS). [28] The CV values found in the present work, for the high, low and high versus low SNR conditions, are similar to those of %fat and (water) T2 relaxation times found in literature and below the general acceptable level of agreement (<10%). This indicates that even in more severely affected patients, simulated by the reduced SNR data-sets, one can accurately quantify PDE-levels. However, the question remains whether this would be sufficient discriminative power to be able to detect potential treatment effects.

### PDE-levels

Longitudinal and spatially resolved ^31^P MRS and qMRI data was used to assess the time course of changes in phosphodiester (PDE)-levels over 2-years in DMD patients, in muscles with different levels of muscle wasting. Our results showed that PDE-levels were significantly elevated in DMD patients compared to controls regardless of the level of muscle wasting at virtually all time-points, and did not change over a two-year time period. This indicates that, although DMD boys were recruited as early as 5.5-year-old, PDE levels were elevated prior to the inclusion of the study. The only muscle that did not show abnormal PDE-levels was the TP muscle at baseline. Interestingly, this muscle is known to be affected relatively late in the course of the disease, which was confirmed in our data by normal %fat at baseline. At later time points, PDE levels became significantly elevated in the TP muscle. This suggests that PDE-levels may increase very early in the disease process, after which they apparently become more stable. This is in agreement with previous work in which PDE-levels of the forearm muscles did not correlate with age in ambulant DMD patients and did not show an increase over one year time.[13, 14] This hypothesis is also strengthened by our own results in the other muscles which did not show a change in PDE-levels over a two-year time period and by the significant correlation with age in the TP muscle at baseline. However, previous work also showed that PDE-levels did correlate with disease progression in non-ambulant patients as well as that PDE-levels showed to be higher in more severely affected patients. [15] Visual inspection of our graphs also suggests a non-linear relation between PDE-levels and age. Consequently, it is likely that PDE-levels do increase with disease progression but much slower than %fat and that a much longer timeframe is needed in order to measure this progression.

PDE-levels in DMD are generally associated with membrane degradation products of the preferentially affected glycolytic Type II fibers.[31] However, the precise origin of this elevation in PDE is not fully understood. Many other diseases have displayed altered intra-cellular PDE-levels in muscle. For instance, in spinal cord injury and congenital lipodystrophy, it has been associated with oxidative stress [32, 33]. In obesity and diabetes altered PDE-levels have been related to reduced mitochondrial capacity [34]. After statin use, in fibromyalgia, Becker muscular dystrophy and facioscapulohumeral muscular dystrophy (FSHD), these alterations have been ascribed to abnormal membrane metabolism associated with muscle dysfunction. [11, 22, 35] In addition, PDE-levels have been shown to be related to a higher content of oxidative type I fibers, to reflect impaired oxidative muscle metabolism, and to increase with respect to age, physical activity level and BMI. [36–38] The total PDE signal measured with ^31^P MRS originates from glycerol 3-phosphocholine (GPC) and glycerol 3-phosphoethanolamine (GPE), which are both related to membrane phospholipid metabolism. [12, 39–41] Recent work showed that GPC, rather than a combination of GPE and GPC, was the main contributor to the changes observed in muscular PDE-levels in obesity and diabetes.[25] GPC has been shown to accumulate due to inhibition of oxidative phosphorylation, which strengthens the relationship with impaired oxidative capacity. [42] Unfortunately, the SNR of our data was too low to ascribe the changes found in myocellular PDE to either GPC or GPE. Although, the exact underlying cause for the elevation in PDE remains unclear and might be multifactorial, this work points out that myocellular PDE-levels detected with ^31^P MRS are extremely interesting in DMD

### Longitudinal evaluation of the other metabolic indices

The majority of the results from the between-group analysis at the three different time-points were in agreement with prior cross-sectional work and have been discussed previously. [10, 15, 43–46] The longitudinal analysis showed that none of the metabolic indices differed between the subsequent time points, which suggests that all metabolic alterations remain stable over a two-year time period. These findings are in agreement with a one year follow-up study in the forearm muscles of DMD.[14] In contrast, the majority of the previous cross-sectional ^31^P MRS studies in more severely affected DMD patients, did show more pronounced metabolic alterations.[10, 15, 44, 45] Similar behavior was visible above the age of 10 in our data, although not reaching statistical significance. Therefore, our results and those of others together suggest that the metabolic indices most likely show a very slow potentially non-linear progression over time after the initial increase. Even though none of the metabolic indices changed over the two-year time period in our study, those metabolic alterations do reflect status of muscle tissue and could therefore provide important insights into the underlying pathophysiology.

### Limitations

Some limitations of the study should be acknowledged. Firstly, some of the MR spectra had to be discarded due to insufficient quality. This could have resulted in some bias towards less severely affected muscles and could possibly explain that no significant abnormalities were found in PCr/ATP ratios. Secondly, the number of patients in the DMD group at 24-month follow-up was relatively low compared to the other time-points. The DMD patients who were unable to participate at 24-month follow-up were generally of older age. Therefore, the results could be biased towards less severely affected muscles and this might have resulted in smaller differences in metabolic indices over the time-points. However, all patients were able to participate in the 12-month follow up and no changes were detected and between baseline and 12-month follow-up. Therefore it seems unlikely that this had a major influence on our results. Thirdly, as the diameter of the muscles changes along the length of the lower leg, it is somewhat difficult to position an individual voxel of the 2D-CSI within an individual muscle over the full length of the coil. The T1-weighted images, obtained as anatomical reference, were carefully used in order to ensure that one individual voxel was located within one individual muscle over the full length of the coil in order to minimize this problem. Fourthly, the fact that DMD boys and HC boys inevitably grow during a 2-year longitudinal study complicates accurate repositioning between subsequent time-points. To circumvent this problem, the T1-weighted images from the previous time-point were used as a visual reference to ensure that 2D-CSI was located at a similar position along the proximo-distal muscle axis. However, the fact that the size of the leg could have increased between the subsequent time points makes it impossible to cover the exact same region. In addition, the SNR of the ^31^P MRS datasets in this work was insufficient to separate GPC and GPE. Previous work showed that, in order to reach sufficient SNR, surface coil localized measurements are necessary. However, with those measurements it is impossible to obtain muscle specific measurements, which is essential in DMD as muscles become affected at different time points and with different rates. Lastly, the relation between SNR and quantification of PDE-levels was only assessed for the TA muscle and in one of the subjects. Therefore, it is possible that this relation is different in the other lower leg muscles. However, this is highly unlikely as accuracy of quantification depends on data-quality and not muscle-type.

### Conclusion

The two-fold elevation in PDE-levels compared to controls detected prior to structural changes and the high reproducibility of the measurements in low and high SNR conditions confirm the potential of PDE as a marker for muscle tissue changes in DMD patients.

## Supporting information

S1 File Example of the planning of the ^31^P 2D-CSI dataset superimposed on a axial T1-weighted image. The spectroscopy grid was positioned in such a way that individual voxels were located in individual muscle over the entire length of the coil: the first, the middle and last slice of the of the T1-weighted image with the voxels placed in the TA, TP, SOL and muscles.
(TIF)

S2 File The complete dataset containing all ^31^P spectra for the 6 analyzed muscles at baseline, 12-month follow-up and 24-month follow-up as well as the ^31^P spectra of the healthy volunteers used for the quality control assesment. (XLSX)
