# Supplementary figures and images for: Spatially localized phosphorous metabolism of skeletal muscle in Duchenne muscular dystrophy patients: 24–month follow-up
Source: PLoS One. 2017 Aug 1;12(8):e0182086. doi: 10.1371/journal.pone.0182086 (PMC5538641; doi:10.1371/journal.pone.0182086)

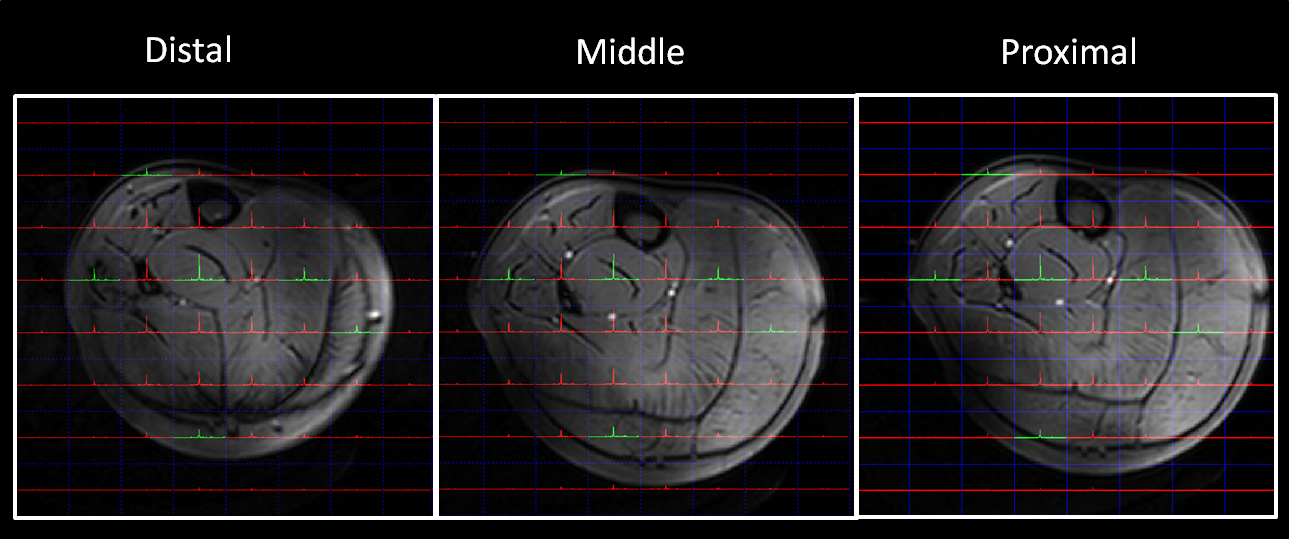

Supplement: S1 File — The spectroscopy grid was positioned in such a way that individual voxels were located in individual muscle over the entire length of the coil: the first, the middle and last slice of the of the T1-weighted image with the voxels placed in the TA, TP, SOL and muscles. (TIF) [file pone.0182086.s001.tif]
